# Performance of radiomics models derived from different CT reconstruction parameters for lung cancer risk prediction

**DOI:** 10.1186/s12890-023-02366-y

**Published:** 2023-04-20

**Authors:** Rui Zhang, Jie Shi, Siyun Liu, Bojiang Chen, Weimin Li

**Affiliations:** 1 Department of Pulmonary and Critical Care Medicine, West China Hospital, Sichuan University, 37 GuoXue Alley, Wuhou District, Chengdu, Sichuan Province 610041 People’s Republic of China; 2 Department of General Practice, West China Hospital, Sichuan University, Chengdu, People’s Republic of China; 3 GE Healthcare, Shanghai, China

**Keywords:** Pulmonary nodules, Radiomics, Computed tomography, Reconstruction parameters

## Abstract

**Background:**

This study analysed the performance of radiomics features extracted from computed tomography (CT) images with different reconstruction parameters in differentiating malignant and benign pulmonary nodules.

**Methods:**

We evaluated routine chest CT images acquired from 148 participants with pulmonary nodules, which were pathologically diagnosed during surgery in West China Hospital, including a 5 mm unenhanced lung window, a 5 mm unenhanced mediastinal window, a 5 mm contrast-enhanced mediastinal window and a 1 mm unenhanced lung window. The pulmonary nodules were segmented, and 1409 radiomics features were extracted for each window. Then, we created 15 cohorts consisting of single windows or multiple windows. Univariate correlation analysis and principal component analysis were performed to select the features, and logistic regression analysis was performed to establish models for each cohort. The area under the curve (AUC) was applied to compare model performance.

**Results:**

There were 75 benign and 73 malignant pulmonary nodules, with mean diameters of 18.63 and 19.86 mm, respectively. For the single-window setting, the AUCs of the radiomics model from the 5 mm unenhanced lung window, 5 mm unenhanced mediastinal window, 5 mm contrast-enhanced mediastinal window and 1 mm unenhanced lung window were 0.771, 0.808, 0.750, and 0.771 in the training set and 0.711, 0.709, 0.684, and 0.674 in the test set, respectively. Regarding the multiple-window setting, the radiomics model based on all four windows showed an AUC of 0.825 in the training set and 0.743 in the test set. Statistically, the 15 models demonstrated comparable performances (P > 0.05).

**Conclusion:**

A single chest CT window was acceptable in predicting the malignancy of pulmonary nodules, and additional windows did not statistically improve the performance of the radiomics models. In addition, slice thickness and contrast enhancement did not affect the diagnostic performance.

**Supplementary Information:**

The online version contains supplementary material available at 10.1186/s12890-023-02366-y.

## Introduction

Pulmonary nodules are commonly detected on computed tomography (CT) of the chest [1]. About 95% of detected pulmonary nodules are benign and have a wide variety of causes (most often granulomas or intrapulmonary lymph nodes), while a small number of nodules are early lung cancers [2, 3]. The optimal diagnostic approach for pulmonary nodules should facilitate timely and effective curative treatment for lung cancer and simultaneously avoid harmful interventions in benign disease [1]. At present, most guidelines recommend noninvasive risk assessment of pulmonary nodules; and CT surveillance is recommended for low-risk nodules, while PET-CT or biopsy or excision is suggested for high-risk nodules [1, 4, 5]. The recommended risk assessment models mainly include the Brock model and Mayo model, which were established based on clinical variables [6, 7].

Nevertheless, drastic increases in computational power and memory have enabled the development and implementation of artificial intelligence techniques in the medical field [8]. For example, radiomics analysis, which can noninvasively mine high-throughput quantitative image features from standard-of-care medical imaging, is gaining incremental importance in cancer research [9]. Most studies have demonstrated that radiomics models could be effective supplementary tools for the decision-making of clinicians in the diagnosis and treatment of cancer, and radiomics models can predict the malignancy of lesions, gene mutation, pathological type, clinical stage, treatment response and prognosis [10]. For pulmonary nodules, previous studies have shown that radiomics models performed well in predicting the lung cancer risk of nodules based on CT images, such as low-dose or routine CT images [11, 12] and unenhanced or contrast-enhanced CT images [13, 14].

However, it was reported that image acquisition and reconstruction parameters such as contrast enhancement, slice thickness, convolution algorithm, tube voltage and current could affect the reproducibility of radiomic features and influence the diagnostic performance of radiomics models [15–17]. For example, Stefano et al. revealed that the diagnostic value of histogram features could vary at different Hounsfield units (HU) [18]. Nevertheless, only one or a few reconstruction parameters were investigated in previous studies of lung cancer risk prediction. The impact of different reconstruction parameters on radiomics feature extraction and analysis has not yet been thoroughly explored.

Hence, this study intended to simultaneously assess how four common chest CT reconstruction windows affect the performance of radiomics features in differentiating malignant and benign pulmonary nodules, which included a 5 mm unenhanced lung window, a 5 mm unenhanced mediastinal window, a 5 mm contrast-enhanced mediastinal window and a 1 mm unenhanced lung window.

## Methods

### Study patients

This retrospective, single-centre study was approved by the institutional review board of the West China Hospital of Sichuan University, and the requirement for informed consent was waived as the privacy and identity information of the participants were protected. The study selected eligible patients discharged from West China Hospital of Sichuan University from 2010 to 2018 based on the following inclusion criteria: (1) the patient was older than 18 years old; (2) there was a solitary pulmonary nodule detected on routine chest CT in the institution; (3) the patient was treated with surgery without receiving chemo- or radiotherapy, and the nodule was pathologically confirmed as primary lung cancer or benign pulmonary lesion; and (4) four chest CT reconstruction windows were available, including a 5 mm unenhanced lung window, a 5 mm unenhanced mediastinal window, a 5 mm contrast-enhanced mediastinal window and a 1 mm unenhanced lung window. Otherwise, the subjects were excluded if (1) the patient was receiving treatment due to other malignancies; (2) the pulmonary nodule was calcified; or (3) the entire nodule volume was not completely shown on all four windows. As the 1 mm contrast-enhanced mediastinal window was only available in a small number of patents, this window was not collected and analysed in the current study. In total, 148 patients with 592 chest CT images were finally enrolled. Clinical variables, including sex, age, and pathological diagnosis, were collected for further analysis.

### CT acquisition protocol

Images were acquired from one 64-slice multidetector CT scanner of the chest (SOMATOM, Definition Flash, Siemens). The scan parameters included: voltage, 80–140 kVp; current, 98–678 mAs; rotation time, 0.5 s; matrix, 512 × 512; table speed, 30.6-139.3 mm/s; reconstruction thickness, 1 and 5 mm. After plain CT, arterial-phase enhanced scans were performed 40 s after intravenous injection of nonionic iodinated contrast medium (300 mg/mL), which was administered for all patients at a dose of 2 mL/kg body weight and rate of 3.5-4.0 mL/s using a power injector from Bayer. Finally, four CT series were reconstructed: 5 mm unenhanced lung window, 5 mm unenhanced mediastinal window, 5 mm contrast-enhanced mediastinal window and 1 mm unenhanced lung window. All images were exported in DICOM format for subsequent analysis.

### CT analysis

Two respiratory physicians (CBJ and ZR, with 10 and 6 years of experience in chest image interpretation, respectively) independently reviewed all the CT images and resolved discrepancies by discussion. All images were reviewed at both lung (width = 1500 HU; level = − 700 HU) and mediastinal (width = 350 HU; level = 40 HU) settings. Nodule diameter, location, texture, consolidation/tumour ratio, spiculation, lobulation and cavity were evaluated on the axial plane of a 1 mm unenhanced lung window as morphological features. The consolidation/tumour ratio, namely, the ratio of the maximum consolidation diameter to the maximum nodule diameter, was calculated to assess the proportion of the solid component [19].

### Image preprocessing, nodule segmentation and radiomics feature extraction

**Fig. 1:**
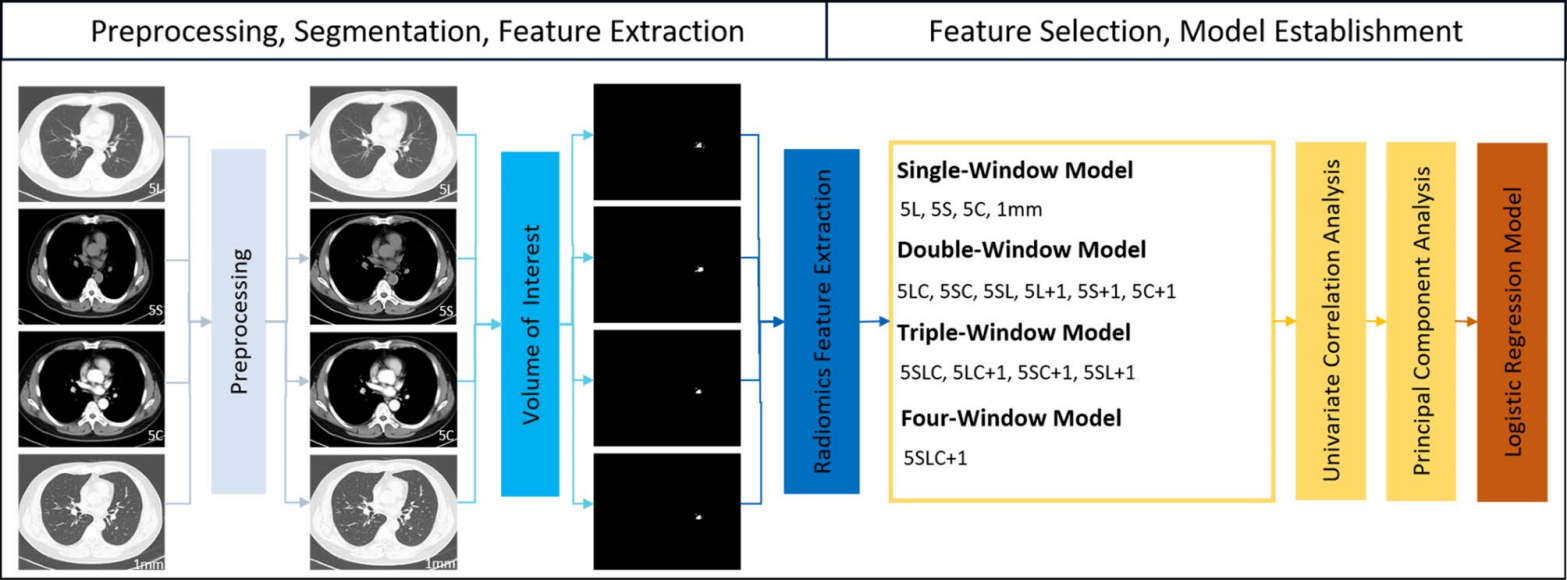
The pipeline of the whole study. 5 L, 5 mm unenhanced lung window; 5 S, 5 mm unenhanced mediastinal window, 5 C, 5 mm contrast-enhanced mediastinal window; 1 mm, 1 mm unenhanced lung window; other model symbols being combinations of single-window abbreviations

**Fig. 2:**
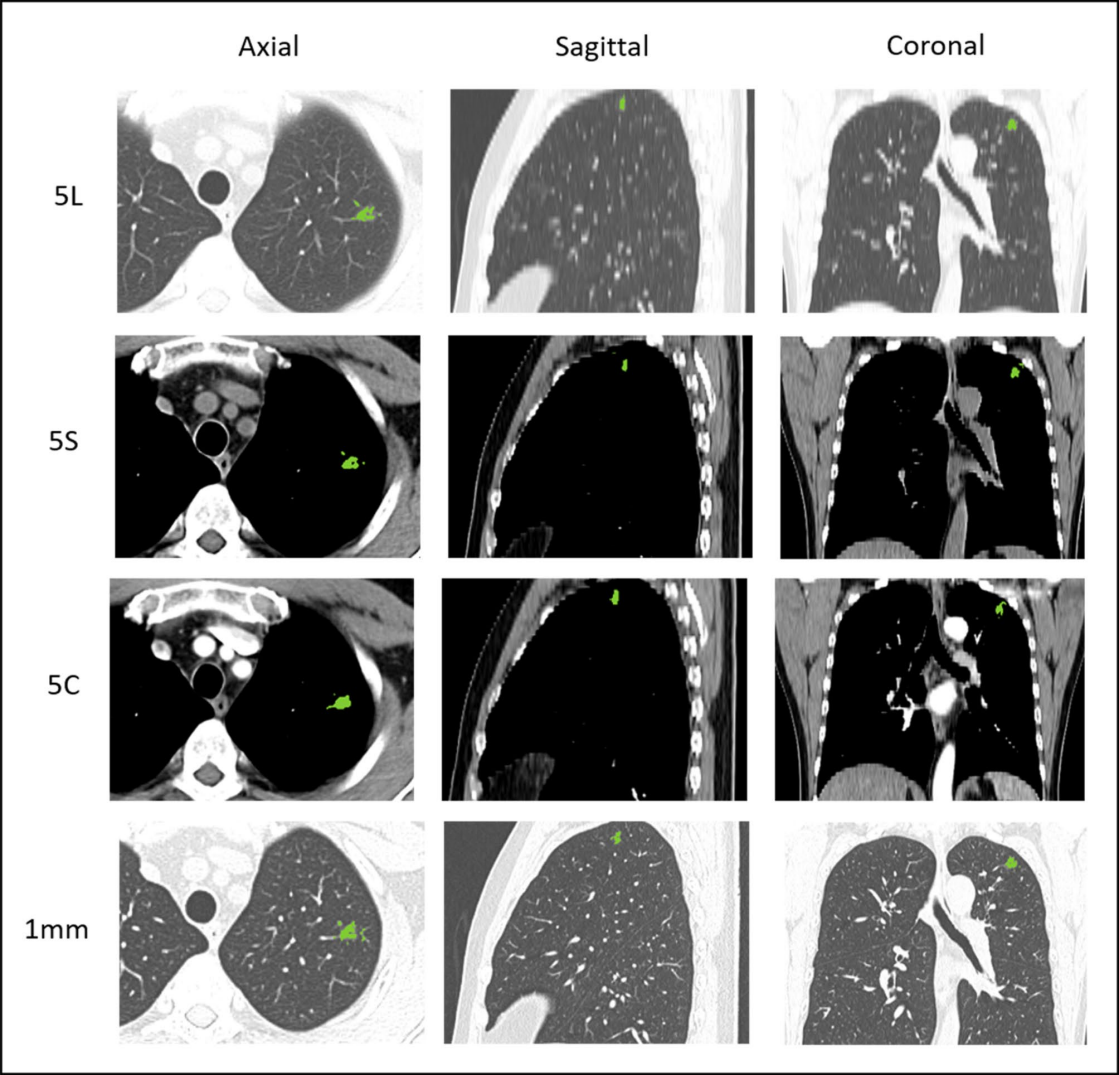
One example of a segmented pulmonary nodule on four windows. 5 L, 5 mm unenhanced lung window; 5 S, 5 mm unenhanced mediastinal window, 5 C, 5 mm contrast-enhanced mediastinal window; 1 mm, 1 mm unenhanced lung window

Figure 1 shows the whole modelling pipeline. First, LK software (Lung Intelligence Kit, version 1.5.0, GE Healthcare) was applied to preprocess images and segment the volume of interest (VOI). All images were preprocessed by resampling (spatial resolution = 1 mm × 1 mm × 1 mm), Laplace enhancement and Gaussian filtering (standard deviation, SD = 0.5). Then, the LK software automatically detected and segmented the whole nodule volume on each reconstruction window, which was accompanied by manual correction (by CBJ and ZR) to ensure that every nodule was accurately delineated. Approximately 40% of the nodules segmented by the software were manually modified slice by slice. One example of a segmented nodule on four windows is shown in Fig. 2.

Subsequently, AK software (Artificial Intelligence Kit, version 3.3.0, GE Healthcare) was applied to automatically extract 1409 three-dimensional radiomics features from each window for all patients, including shape features (n = 14), first-order features (n = 18), grey level cooccurrence matrix features (n = 24), grey level run length matrix features (n = 16), grey level size zone matrix features (n = 16), neighbouring grey tone difference matrix features (n = 5), grey level dependence matrix features (n = 14), Laplacian of Gaussian filtered features (n = 186, Sigma = 1.0, 3.0, 5.0), first-order wavelet-filtered features (n = 744, Level = 1) and 3-D local binary pattern related features (n = 372, Level = 2, Radius = 1.00, Subdivision = 1).

### Feature selection and model construction

To evaluate the diagnostic value of radiomics features derived from independent reconstruction windows as well as multiple reconstruction windows, we regrouped the four windows into 15 cohorts to establish 15 models, including four single-window models, six double-window models, four triple-window models and one four-window model. All subjects were randomly separated into the training set (N = 104) and test set (N = 44) at a ratio of 7:3.

As there were thousands of radiomics features in each cohort, feature selection was performed to identify representative features and avoid overfitting. Specifically, after standardization with Z score transformation, univariate correlation analysis (cut-off = 0.7) and principal component analysis (number of components = 10) were performed to select features. In the study, the number of principal components was set to ten based on the scree diagram derived from Kaiser‒Meyer‒Olkin analysis. Finally, the logistic regression model was selected and established in the training set and validated in the test set for each cohort after comparison with the other machine learning models (support vector machine, decision tree, decision forest, Bayes, K-nearest neighbour).

### Statistical analysis

The continuous variables are described as the mean ± SD and were compared with Student’s t test, while the categorical variables are described as the number of cases (proportion) and were compared with the chi-square test or Fisher’s exact test. In addition, the receiver operating characteristic (ROC) curve and area under the curve (AUC) were acquired to evaluate the discrimination performance of the models. Delong’s test was conducted to compare AUCs. The accuracy, sensitivity and specificity were calculated based on Youden’s J index. Moreover, the Hosmer‒Lemeshow test was applied to illustrate the goodness of fit, and decision curve analysis was conducted to evaluate the clinical usefulness of the established models. A two-tailed p value < 0.05 indicated statistical significance. All statistical analyses were implemented using the Institute of Precision Medicine Statistics (version 1.1, GE Healthcare) and SPSS (version 28.0).

## Results

### Clinical characteristics

The clinical characteristics of the enrolled patients are summarized in Table 1. Among all 148 patients, the age ranged from 26 to 77 years old, with a mean ± SD of 54.49 ± 11.1 years. The patients with malignant nodules were older (57.12 ± 10.56 vs. 51.92 ± 11.05 years, P = 0.004). There were 57 males and 91 females, and no significant difference was observed between the malignant and benign groups (P = 0.293).

**Table 1:** Clinical characteristics of enrolled patients

Characteristics	Total (N = 148)	Malignant group (N = 73)	Benign group (N = 75)	P value
Age, mean ± SD, year	54.49 ± 11.10	57.12 ± 10.56	51.92 ± 11.05	0.004
Sex, n (%)				0.293
Male	57 (38.51)	25 (34.25)	32 (42.67)	
Female	91 (61.49)	48 (65.75)	43 (57.33)	
Nodule diameter, mean ± SD, mm	19.15 ± 17.63	19.86 ± 18.61	18.63 ± 16.78	0.672
Nodule location, n (%)				0.953
Upper left lobe	37 (25.00)	17 (23.29)	20 (26.67)	
Upper right lobe	51 (34.46)	27 (36.99)	24 (32.00)	
Lower left lobe	24 (16.22)	11 (15.07)	13 (17.33)	
Lower right lobe	25 (16.89)	13 (17.80)	12 (16.00)	
Middle right lobe	11 (7.43)	5 (6.85)	6 (8.00)	
Nodule texture, n (%)				< 0.001
Solid	79 (53.38)	22 (30.14)	57 (76.00)	
Subsolid	69 (46.62)	51 (69.86)	18 (24.00)	
Consolidation/tumor ratio, n (%)				< 0.001
> 0.5	98 (66.22)	36 (49.32)	62 (82.67)	
≤ 0.5	50 (33.78)	37 (50.68)	13 (17.33)	
Spiculation, n (%)				0.669
Yes	45 (30.41)	21 (28.77)	24 (32.00)	
No	103 (69.59)	52 (71.23)	51 (68.00)	
Lobulation, n (%)				0.774
Yes	45 (30.41)	23 (31.51)	22 (29.33)	
No	103 (69.59)	50 (68.49)	53 (70.67)	
Cavity, n (%)				0.095*
Yes	9 (6.08)	7 (9.59)	2 (2.67)	
No	139 (93.92)	66 (90.41)	73 (97.33)	

* The P value was calculated using Fisher’s exact test

Abbreviations: SD, standard deviation

Regarding the pathology of the enrolled subjects, 75 (50.68%) nodules were benign, and 73 (49.32%) nodules were malignant. The malignant nodules included adenocarcinomas (N = 68, 93.15%) and squamous carcinomas (N = 5, 6.85%), whereas the benign nodules consisted of chronic inflammatory lesions (N = 30, 40.00%), granulomas (N = 15, 20.00%), hamartomas (N = 11, 14.67%), tuberculosis (N = 8, 10.67%) and so on. Detailed information on the pathological diagnosis of the pulmonary nodules is summarized in Table S1.

In terms of the morphological features of the enrolled malignant and benign nodules, the mean diameters were 19.15 mm and 18.63 mm, respectively (P = 0.672), and most were located in the right upper lobe (36.99% vs. 32.00%, P = 0.953). There were many more subsolid nodules in the malignant group (69.86% vs. 24.00%, P < 0.001). However, no significant difference was observed regarding spiculation (28.77% vs. 32.00%, P = 0.669), lobulation (31.51% vs. 29.33%, P = 0.774) and cavity sign (9.59% vs. 2.67%, P = 0.095) between the malignant and benign groups.

### Radiomics feature selection

When the four windows were regrouped into 15 cohorts, there were 1409 features for the single-window cohort, 2818 features for the double-window cohort, 4227 features for the triple-window cohort and 5636 features for the four-window cohort. After univariate correlation analysis, approximately 10.8% ± 1% radiomics features remained in each cohort. Then, the remaining features were compressed to 10 representative components by principal component analysis to establish models for each cohort. Details of the radiomics feature selection are demonstrated in Table S2.

### Performance of radiomics models

**Fig. 3:**
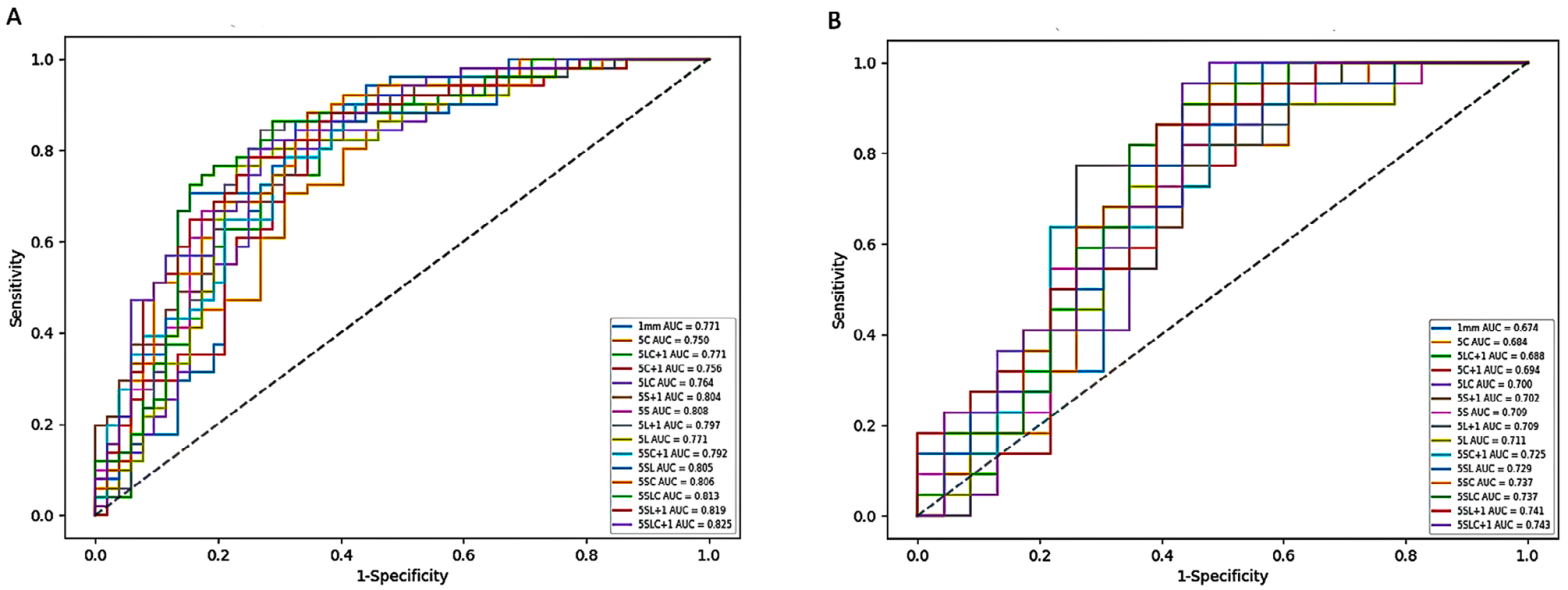
The ROC curves of all models in the training set (A) and test set (B). 5 L, 5 mm unenhanced lung window; 5 S, 5 mm unenhanced mediastinal window, 5 C, 5 mm contrast-enhanced mediastinal window; 1 mm, 1 mm unenhanced lung window; other model symbols being combinations of single-window abbreviations

Figure 3 shows the ROC curves of the 15 established models, and Table 2 summarizes the corresponding diagnostic values. There were 11 models with an AUC greater than 0.700 in the test set. However, no significant differences were observed among the AUCs by DeLong’s tests (Table S3). Taken as a whole, all models demonstrated similar performance.

**Table 2:** The predictive performance of 15 radiomics models

Models	Training set				Test set				P value*
	Accuracy	AUC (95% CI)	Sensitivity	Specificity	Accuracy	AUC (95% CI)	Sensitivity	Specificity	
**5SLC + 1**	0.767	0.825 (0.756, 0.888)	0.784	0.75	0.644	**0.743 (0.607, 0.862)**	0.682	0.609	0.1443
**5SL + 1**	0.757	0.819 (0.745, 0.883)	0.765	0.75	0.644	**0.741 (0.611, 0.858)**	0.682	0.609	0.3137
**5SLC**	0.777	0.813 (0.736, 0.883)	0.843	0.712	0.711	**0.737 (0.601, 0.859)**	0.773	0.652	0.0578
**5SC**	0.757	0.806 (0.732, 0.874)	0.863	0.654	0.711	**0.737 (0.599, 0.860)**	0.864	0.565	0.1213
**5SL**	0.767	0.805 (0.729, 0.873)	0.686	0.846	0.644	**0.729 (0.597, 0.852)**	0.591	**0.696**	0.1547
**5SC + 1**	0.728	0.792 (0.718, 0.862)	0.902	0.558	**0.733**	**0.725 (0.584, 0.849)**	**0.955**	0.522	0.0705
**5 L**	0.757	0.771 (0.690, 0.847)	0.745	0.769	0.689	**0.711 (0.570, 0.840)**	0.727	0.652	0.3912
**5 L + 1**	0.777	0.797 (0.719, 0.867)	0.824	0.731	0.667	**0.709 (0.564, 0.840)**	0.773	0.565	0.0233
**5 S**	0.757	0.808 (0.735, 0.877)	0.784	0.731	0.644	**0.709 (0.567, 0.830)**	0.636	0.652	0.0554
**5 S + 1**	0.738	0.804 (0.731, 0.871)	0.804	0.673	0.6	**0.702 (0.558, 0.826)**	0.636	0.565	0.0058
**5LC**	0.757	0.764 (0.683, 0.840)	0.784	0.731	0.622	**0.700 (0.556, 0.830)**	0.591	0.652	0.1426
**5 C + 1**	0.728	0.756 (0.673, 0.831)	0.824	0.635	0.622	0.694 (0.543, 0.828)	0.773	0.478	0.0776
**5LC + 1**	0.728	0.771 (0.693, 0.845)	0.843	0.615	0.644	0.688 (0.542, 0.819)	0.818	0.478	0.1569
**5 C**	0.689	0.750 (0.670, 0.827)	0.863	0.519	0.622	0.684 (0.540, 0.814)	**0.955**	0.304	0.4862
**1 mm**	0.748	0.771 (0.682, 0.846)	0.804	0.692	0.644	0.674 (0.528, 0.802)	0.682	0.609	0.0389

* The P value was calculated using Hosmer-Lemeshow Test from the test set. When P > 0.05, the model had a high goodness of fit

Abbreviations: AUC, area under the curve; 5 L, 5 mm unenhanced lung window; 5 S, 5 mm unenhanced mediastinal window, 5 C, 5 mm contrast-enhanced mediastinal window; 1 mm, 1 mm unenhanced lung window. Other model symbols were combinations of single-window abbreviations, for example, 5SLC + 1 = 5 mm unenhanced mediastinal window + 5 mm unenhanced lung window + 5 mm contrast-enhanced mediastinal window + 1 mm unenhanced lung window

In detail, the four-window model demonstrated an AUC of 0.743 (95% CI, 0.607–0.862), an accuracy of 0.644, a sensitivity of 0.682 and a specificity of 0.609. For the single-window models, first, the 5 mm unenhanced-lung-window model showed an AUC of 0.711 (95% CI, 0.570, 0.840), an accuracy of 0.689, a sensitivity of 0.727 and a specificity of 0.652. Second, the 5 mm unenhanced mediastinal window model showed an AUC of 0.709 (95% CI, 0.567, 0.830), an accuracy of 0.644, a sensitivity of 0.636 and a specificity of 0.652. Third, the 5 mm contrast-enhanced mediastinal window model demonstrated an AUC of 0.684 (95% CI, 0.540, 0.814), an accuracy of 0.622, a sensitivity of 0.955 and a specificity of 0.304. Finally, the 1 mm unenhanced-lung-window model had an AUC of 0.674 (95% CI, 0.528, 0.802), an accuracy of 0.644, a sensitivity of 0.682 and a specificity of 0.609. The mentioned diagnostic values were from the test set.

**Fig. 4:**
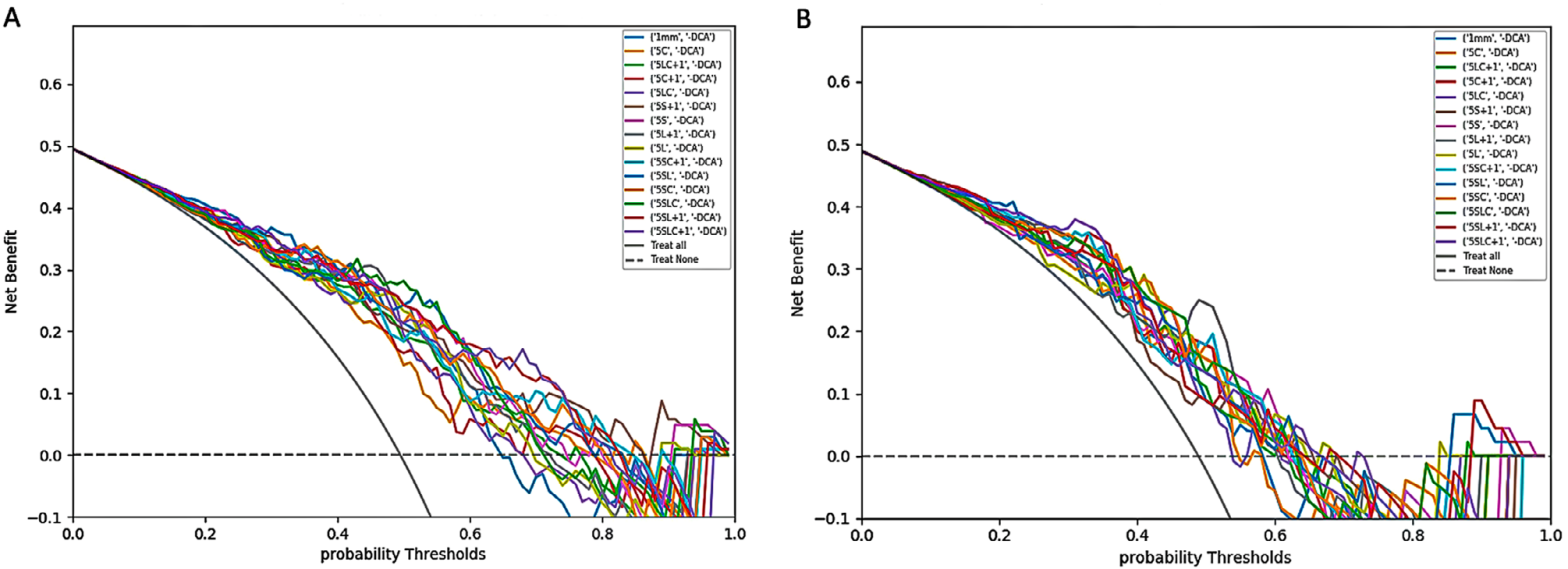
The decision curve analyses for all models in the training set (A) and test set (B). 5 L, 5 mm unenhanced lung window; 5 S, 5 mm unenhanced mediastinal window, 5 C, 5 mm contrast-enhanced mediastinal window; 1 mm, 1 mm unenhanced lung window; other model symbols being combinations of single-window abbreviations

As shown in Table 2, the values predicted by most models matched well with the actual data (Hosmer‒Lemeshow Test, P > 0.05). Figure 4 demonstrates the decision curve analyses for 15 models, which indicated that the established radiomics models had potential clinical practicability to some degree.

## Discussion

In the current study, by regrouping four chest CT reconstruction windows, a total of 15 single-window and multiple-window radiomics models were established. The results indicated that all models demonstrated similar performance in differentiating malignant and benign pulmonary nodules.

Some multiple-window radiomics models demonstrated higher AUCs than single-window models, but no significant differences were observed. Our results are consistent with those of Yang et al., who showed that radiomics signatures from both plain and vein-phase CT images were not superior to either plain signatures or vein signatures in differentiating solitary granulomas and solid lung adenocarcinomas [14]. However, two recent studies on lung cancer indicated that multiple-window-based models performed better than single-window-based models [20, 21]. For example, Lu et al. found that when predicting lung tumour growth patterns from radiomics features, the models constructed based on the lung window or the difference region (subtracting the mediastinal window region from the lung window region) were inferior to the model established based on both of them [21]. A possible explanation for these findings is that different strategies were applied in acquiring VOIs, and different kinds of features were used in the models.

In our investigations of diagnostic prediction, thinner reconstruction slice thickness did not provide added diagnostic information. Similarly, Park et al. found that the performance of radiomics models in prognostic prediction was not significantly changed when they were applied to 1-, 3-, and 5-mm slice thickness datasets [22]. Nevertheless, He et al. showed that thin-section CT-based radiomics signatures indeed had better diagnostic performance for solitary pulmonary nodules [23]. Our unexpected results may be explained by the larger nodule diameters of the enrolled patients (mean, 19.15 mm). As shown in one recent study, CT reconstruction parameters (including slice thickness) did not substantially affect the diagnostic performance of radiomics signatures for pulmonary nodules larger than 10 mm, but it mattered for pulmonary nodules less than 10 mm, where thin-section CT-based radiomics signatures performed better [24]. This study pointed out that for nodules greater than 10 mm, perhaps there is an optimal amount of imaging slices at which further addition will no longer increase diagnostic efficacy and may even decrease performance due to increased noise [24].

It is controversial how contrast enhancement affects radiomics analysis in predicting the malignancy of pulmonary nodules. He et al. showed that radiomics signatures from unenhanced CT demonstrated better discrimination and classification capability, as the biological heterogeneity within the tumour may be confounded by the contrast material [23]. On the other hand, Wu et al. found that contrast enhancement did not impact the utility of radiomics analysis, and the hypothesis was that the selected radiomics features measure the correlation, uniformity, and deviation of the pixels [13]. Nevertheless, our results indicated that radiomics signatures from the unenhanced and contrast-enhanced CT images had comparable performance in lung cancer risk prediction, where no significant difference was observed.

There were some limitations in the current study. First, this was a retrospective, single-centre study with a relatively small population. Second, each regrouped cohort had a large number of features, at least 1,409, which could cause overfitting of the model. However, after trying as many feature selection methods and machine learning models as possible, we believe that the methods finally used were relatively optimal. Finally, all CT images were acquired from one 64-slice multidetector CT scanner of the chest. In addition, most centres may no longer use 5 mm reconstruction, as chest high-resolution CT is widely considered more accurate in the assessment of pulmonary nodules. Additionally, contrast-enhanced CT is not recommended for nodule evaluation unless certain conditions apply. Therefore, these aspects may limit the generalizability of our results.

In the future, we will carry out prospective studies in a clinical setting to further validate the current results. Afterwards, the software used in our study can be optimized and installed in the work environment of physicians and radiologists to facilitate the use of proposed radiomics models in daily practice and to see if the established models could improve their diagnostic efficacy. Based on the specific design of the current study, the potential application of our results in clinical practice may be limited to patients with pulmonary nodules who may need hospitalization.

## Conclusion

The current study evaluated the performance of radiomics features extracted from routine chest CT images with different reconstruction parameters in differentiating malignant and benign pulmonary nodules. The results indicated that radiomics features obtained from multiple windows demonstrated comparable performance to those from single windows. In addition, slice thickness and contrast enhancement did not substantially affect the diagnostic performance.

## Electronic supplementary material

Below is the link to the electronic supplementary material.

Supplementary Material 1

## Data Availability

Please contact author (Weimin Li) for data requests.

## Abbreviations

CT: Computed tomography
HU: Hounsfield unit
VOI: Volume of interest
SD: Standard deviation
ROC curve: Receiver operating characteristic curve
AUC: Area under the curve
