# Supplementary material for: Performance of radiomics models derived from different CT reconstruction parameters for lung cancer risk prediction
Source: BMC Pulm Med. 2023 Apr 20;23:132. doi: 10.1186/s12890-023-02366-y (PMC10116652; doi:10.1186/s12890-023-02366-y)
Supplement: Supplementary file 1 — Supplementary Material 1 [file 12890_2023_2366_MOESM1_ESM.docx]

**Table S1 Pathological diagnosis of enrolled pulmonary nodules**

| Pathology | Number | Percentage (%) |
| --- | --- | --- |
| Malignant nodule |  |  |
| Adenocarcinoma | 68 | 93.15 |
| Squamous carcinoma | 5 | 6.85 |
| Total | 73 | 100.00 |
| Benign nodule |  |  |
| Chronic inflammatory lesion | 30 | 40.00 |
| Granuloma | 15 | 20.00 |
| Hamartoma | 11 | 14.67 |
| Tuberculosis | 8 | 10.67 |
| Precursor glandular lesion | 7 | 9.33 |
| Others | 4 | 5.33 |
| Total | 75 | 100.00 |

**Table S2 Radiomics feature selection**

| **Models** | **No. of Original Features** | **Univariate correlation analysis** | | **Principal component analysis** |
| --- | --- | --- | --- | --- |
|  |  | **No. of selected features** | **Proportion** | **No. of components** |
| **5L** | 1409 | 183 | 12.99% | 10 |
| **5S** | 1409 | 172 | 12.21% | 10 |
| **5C** | 1409 | 173 | 12.28% | 10 |
| **1mm** | 1409 | 135 | 9.58% | 10 |
| **5LC** | 2818 | 321 | 11.39% | 10 |
| **5SC** | 2818 | 309 | 10.97% | 10 |
| **5SL** | 2818 | 311 | 11.04% | 10 |
| **5L+1** | 2818 | 297 | 10.54% | 10 |
| **5S+1** | 2818 | 278 | 9.87% | 10 |
| **5C+1** | 2818 | 292 | 10.36% | 10 |
| **5SLC** | 4227 | 448 | 10.60% | 10 |
| **5LC+1** | 4227 | 435 | 10.29% | 10 |
| **5SC+1** | 4227 | 425 | 10.05% | 10 |
| **5SL+1** | 4227 | 422 | 9.98% | 10 |
| **5SLC+1** | 5636 | 563 | 9.99% | 10 |

Abbreviations: 5L, 5 mm unenhanced lung window; 5S, 5 mm unenhanced mediastinal window, 5C, 5 mm contrast-enhanced mediastinal window; 1mm, 1 mm unenhanced lung window. Other model symbols were combinations of single-window abbreviations, for example, 5SLC+1 = 5 mm unenhanced mediastinal window + 5 mm unenhanced lung window + 5 mm contrast-enhanced mediastinal window + 1 mm unenhanced lung window.

**Table S3** **Delong’s test results in testing set**

| **Models** | **1mm** | **5C** | **5LC+1** | **5C+1** | **5LC** | **5S+1** | **5S** | **5L+1** | **5L** | **5SC+1** | **5SL** | **5SC+1** | **5SLC** | **5SL+1** | **5SLC+1** |
| --- | --- | --- | --- | --- | --- | --- | --- | --- | --- | --- | --- | --- | --- | --- | --- |
| **1mm** | 1.000 | 0.882 | 0.742 | 0.568 | 0.723 | 0.503 | 0.590 | 0.450 | 0.657 | 0.210 | 0.449 | 0.324 | 0.376 | 0.212 | 0.185 |
| **5C** | 0.882 | 1.000 | 0.946 | 0.844 | 0.779 | 0.778 | 0.700 | 0.640 | 0.690 | 0.382 | 0.504 | 0.310 | 0.384 | 0.379 | 0.310 |
| **5LC+1** | 0.742 | 0.946 | 1.000 | 0.833 | 0.794 | 0.664 | 0.681 | 0.419 | 0.692 | 0.255 | 0.392 | 0.276 | 0.299 | 0.089 | 0.055 |
| **5C+1** | 0.568 | 0.844 | 0.833 | 1.000 | 0.915 | 0.832 | 0.785 | 0.617 | 0.797 | 0.221 | 0.553 | 0.368 | 0.438 | 0.308 | 0.215 |
| **5LC** | 0.723 | 0.779 | 0.794 | 0.915 | 1.000 | 0.973 | 0.873 | 0.840 | 0.829 | 0.647 | 0.554 | 0.421 | 0.398 | 0.429 | 0.390 |
| **5S+1** | 0.503 | 0.778 | 0.664 | 0.832 | 0.973 | 1.000 | 0.884 | 0.856 | 0.887 | 0.401 | 0.584 | 0.460 | 0.477 | 0.148 | 0.084 |
| **5S** | 0.590 | 0.700 | 0.681 | 0.785 | 0.873 | 0.884 | 1.000 | 1.000 | 0.974 | 0.757 | 0.578 | 0.484 | 0.546 | 0.511 | 0.489 |
| **5L+1** | 0.450 | 0.640 | 0.419 | 0.617 | 0.840 | 0.856 | 1.000 | 1.000 | 0.970 | 0.692 | 0.676 | 0.543 | 0.593 | 0.422 | 0.388 |
| **5L** | 0.657 | 0.690 | 0.692 | 0.797 | 0.829 | 0.887 | 0.974 | 0.970 | 1.000 | 0.838 | 0.703 | 0.693 | 0.682 | 0.592 | 0.594 |
| **5SC+1** | 0.210 | 0.382 | 0.255 | 0.221 | 0.647 | 0.401 | 0.757 | 0.692 | 0.838 | 1.000 | 0.940 | 0.757 | 0.800 | 0.680 | 0.541 |
| **5SL** | 0.449 | 0.504 | 0.392 | 0.553 | 0.554 | 0.584 | 0.578 | 0.676 | 0.703 | 0.940 | 1.000 | 0.830 | 0.797 | 0.738 | 0.730 |
| **5SC+1** | 0.324 | 0.310 | 0.276 | 0.368 | 0.421 | 0.460 | 0.484 | 0.543 | 0.693 | 0.757 | 0.830 | 1.000 | 1.000 | 0.927 | 0.869 |
| **5SLC** | 0.376 | 0.384 | 0.299 | 0.438 | 0.398 | 0.477 | 0.546 | 0.593 | 0.682 | 0.800 | 0.797 | 1.000 | 1.000 | 0.922 | 0.870 |
| **5SL+1** | 0.212 | 0.379 | 0.089 | 0.308 | 0.429 | 0.148 | 0.511 | 0.422 | 0.592 | 0.680 | 0.738 | 0.927 | 0.922 | 1.000 | 0.926 |
| **5SLC+1** | 0.185 | 0.310 | 0.055 | 0.215 | 0.390 | 0.084 | 0.489 | 0.388 | 0.594 | 0.541 | 0.730 | 0.869 | 0.870 | 0.926 | 1.000 |

Abbreviations: 5L, 5 mm unenhanced lung window; 5S, 5 mm unenhanced mediastinal window, 5C, 5 mm contrast-enhanced mediastinal window; 1mm, 1 mm unenhanced lung window. Other model symbols were combinations of single-window abbreviations, for example, 5SLC+1 = 5 mm unenhanced mediastinal window + 5 mm unenhanced lung window + 5 mm contrast-enhanced mediastinal window + 1 mm unenhanced lung window.
